# Online Tailored Decision Aid for Maternal Pertussis Vaccination in a Randomized Controlled Trial: Process Evaluation Study

**DOI:** 10.2196/50709

**Published:** 2025-07-08

**Authors:** Charlotte Anraad, Pepijn van Empelen, Robert AC Ruiter, Hilde M van Keulen

**Affiliations:** 1 Department of Work & Social Psychology Faculty of Psychology and Neuroscience Maastricht University Maastricht The Netherlands; 2 TNO Child Health Netherlands Organization for Applied Scientific Research Leiden The Netherlands

**Keywords:** decision aid, process evaluation, informed decision making, intervention mapping, vaccine hesitancy, maternal pertussis vaccination

## Abstract

**Background:**

To promote informed decision-making and maternal pertussis vaccination (MPV) uptake, we systematically developed an interactive, web-based decision aid for pregnant users. Intervention reach (the percentage of participants in the intervention group who used the intervention), use (how much and how long those participants used the intervention), and acceptability (how positively they evaluated the intervention) are essential for it to be effective and should be reported to assess which intervention components may have been effective.

**Objective:**

This is a process evaluation aiming to evaluate (1) the reach and (2) the use, and (3) the acceptability of the intervention.

**Methods:**

We analyzed the reach and use of the intervention among participants in the intervention group of a randomized controlled trial (RCT) that assessed the effects of an online tailored decision aid in the form of a web app. Participants were recruited via social media and midwifery clinics and invited via email to use the intervention at 18 weeks of pregnancy. Reach was measured objectively by assessing the number of participants who visited the intervention at least once. Use of the intervention was logged and included time spent on the decision aid, the number of times clicked, pages visited, and answers given in interactive components. Data from the baseline survey (at <18 wk of pregnancy) were used to measure sociodemographics, informed decision-making, MPV uptake, and determinants of uptake. A posttest survey (20-22 weeks of pregnancy) was used to evaluate the acceptability of the decision aid. We report the findings descriptively and assess baseline differences between those who used versus those who did not use the intervention.

**Results:**

Of the 586 participants in the intervention group, 463 (79%) reached the home page of the intervention. Intervention reach appeared higher among those in their first pregnancy (8.35% difference, *P*=.11), those recruited via their midwife rather than via social media (10.56% difference, *P*=.04), and those who had completed a higher educational level (7.35% difference, *P*=.06). On average, participants spent 4.25 (SD 4.39) minutes on the decision aid. Most participants used the decision aid once (56.2% of those who reached it, n=260) or twice (26.6%, n=123). The average number of clicks was 27.24 (SD 25.08) and varied widely. Regarding acceptability, participants evaluated the decision aid positively with an overall grade of 8.0 out of 10 (SD 1.01). In total, 38.9% (180/463) of participants who used the intervention indicated that the decision aid helped them with their MPV decision-making.

**Conclusions:**

The reach of the decision aid was successful with 79%, and participants were very positive about the decision aid. The use of the intervention (eg, time spent on the intervention) leaves room for improvement and should be improved to maximize intervention effects.

## Introduction

In December 2019, maternal immunization during pregnancy (ie, maternal pertussis vaccination [MPV]) was introduced into the National Immunisation Programme (NIP) in the Netherlands. The aim of MPV is to protect infants from pertussis in their first months of life [1]. MPV is given at 22 weeks of pregnancy. Upon introduction in 2019, the uptake of MPVs in the Netherlands was 70% in 2020 [2].

Getting an MPV is voluntary. A decision aid is a tool aimed at preparing people to make a decision [3]. Because decision aids have shown promising results for screening decisions, leading to greater knowledge and lower decisional conflict among users, we have developed an online decision aid for MPV decisions [4-6]. This is the first decision aid for MPV to our knowledge.

In an overarching study (preregistration [7]), we investigated the effects of the intervention on MPV uptake, informed decision-making (IDM), and determinants of uptake [8]. Results showed a positive effect on IDM, and no significant effect on MPV uptake [8]. However, the intervention consists of several components targeting different outcomes and is offered to a varied group of participants, and such complexity requires an evaluation beyond effects only [9]. Therefore, in this study, we aimed to gain a further understanding of the actual usage of the interventions and the extent to which intervention components contributed to the outcomes by means of a process evaluation. A process evaluation is defined as an “assessment of fidelity and quality of implementation, clarifying causal mechanisms and identifying contextual factors associated with variation in outcomes” [10]. A process evaluation can be used for the following three objectives: (1) it provides insight into the extent to which an intervention and its various components are used, to be able to identify effective components and mechanisms [9,11,12]; (2) it provides insight into the implementation of the intervention, thereby avoiding a type 3 error (concluding that an intervention is ineffective due to unsuccessful implementation), and (3) it provides recommendations for intervention improvement [11].

In this study, we focused on reach, use, acceptability, and satisfaction. Reach refers to the proportion of participants who use the interventions when it is offered to them (ie, all participants in the intervention group of the randomized controlled trial [RCT]). Use refers to the extent to which they will use it. Reviews about decision aids often do not report their reach and use [4,5]. This may be because many studies offer decision aids in a controlled environment (where participants use the decision aid at a research location), in which the reach is 100%. In an uncontrolled environment like in our study, studies have reported a reach between 60% and 65% [13,14]. We will assess whether reach and use are related to baseline characteristics and sociodemographics, because we expect that certain subgroups, such as those in doubt about MPV, may have a higher need for the use of the intervention.

## Methods

### Study Design

This study is part of an RCT to assess intervention effectiveness on IDM, MPV uptake, and determinants of MPV uptake [8]. The original protocol has been described previously [7], and the effect evaluation is described in another study [8]. Because we evaluated intervention reach and use, only participants from the intervention condition were included in this study (N=586). The target group of the study was women in the Netherlands who were less than 18 weeks pregnant at the time of inclusion. Other inclusion criteria were a command of the Dutch language and having given informed consent. For this study, only participants who were in the intervention group of the RCT were included. The study took place between November 2020 and April 2022. The RCT protocol has been described elsewhere in detail [8].

### Ethical Considerations

This study was reviewed by the TNO institutional review board for human research (registration number 2018-01). All participants gave informed consent, and the data were pseudonymized. Participants received a voucher for the value of €5 (equivalent to approximately US $5.87 based on the average conversion rate of €1=US $1.174 applicable during the study period; European Central Bank), to thank them for their participation. All the data presented here cannot be linked to individuals.

### Procedure

Participants were invited to participate via midwifery clinics (n=62) and social media (Facebook and Instagram [Meta]). Midwifery clinics were geographically located in different rural and urban areas in the Netherlands. Participants could enroll in the study if they were less than 18 weeks pregnant. We chose this cutoff point because this is generally before participants make an appointment to receive MPV. They could follow a website link in the invitation letter that directed them to a website with study information and an informed consent form. Informed consent meant giving permission to participate in the questionnaires and random assignment to the control or intervention condition, and permission to request vaccination status from Praeventis, which is the national vaccination register. Those who provided informed consent were assigned a unique participant code and were immediately directed to the online baseline questionnaire, which assessed sociodemographics, IDM, and psychological determinants of MPV uptake targeted in the decision aid. Those who did not fill out the baseline questionnaire at 17 weeks of pregnancy received a reminder via email. After filling out the baseline questionnaire, participants were randomized to either of the 2 conditions.

Participants who were recruited online were randomized into the intervention and control conditions. Participants recruited via midwifery clinics were randomized at the clinic level. Participants in the intervention condition received a link via email to the intervention with their personal participant code at 18 weeks of pregnancy. They could visit the intervention as much as they wanted until they received an invitation and link via email to fill out the posttest measurement at 20 weeks of pregnancy. This is generally around the time that pregnant women make an appointment to receive MPV at 22 weeks, or might already have received the vaccination. At posttest measurement, IDM, decisional certainty, and determinants of MPV uptake were measured again, and a subjective evaluation of the intervention was performed. Those who had not filled out the posttest measurement at 21 weeks received a reminder via email. Participants who had, for whatever reason, already received MPV when filling out the posttest measurement were excluded because this may have influenced their answers.

A more extensive description of the procedure can be found in another study [8].

### Intervention

The intervention is an online decision aid. Participants were sent an email with an invitation to use the decision aid at 18 weeks of pregnancy. The decision aid is a mobile-first web application that can be visited on any device or saved as an app on a mobile phone. The systematic development of the decision aid has been described in detail elsewhere [15], but we will provide some information on this below, as it is relevant to this study. We used Intervention Mapping [16], a systematic intervention planning framework, to develop the intervention. As part of these planning activities, we defined behavioral objectives and identified important, changeable behavioral determinants of vaccination uptake. Next, we matched these with behavior change techniques, which were then translated into practical applications. We used user-centered design methods to create the intervention, aiming to meet the needs and user preferences of the target group. We involved the target group in 4 iterations during the development process, with new participants in each round. Nielsen and Landauer [17] determined that 5-8 participants per iteration identified most usability issues. In all pretests, we involved pregnant women of diverse ages and backgrounds. The pretests aimed to get participants’ feedback on the intervention’s clarity, relevance, usability, and overall structure. In the initial pretest, a focus group consisting of 6 pregnant women was presented with a static intervention prototype. In the second iteration, 5 pregnant women individually used an interactive prototype of the intervention during think-aloud sessions. The third iteration featured a full, interactive version of the intervention, with 6 pregnant women using the intervention individually during a think-aloud session. The fourth iteration was a usability test with 4 low-literacy users in individual think-aloud sessions. After the final iteration, the intervention was tested further by members of the project group on various devices to ensure usability. Adjustments made based on each iteration are described in our other paper [15].

This resulted in a decision aid with the aim to actively inform users about MPV, help them process and weigh the information, and deliberate on what the information means for them in the light of their personal values. In addition, the decision aid aimed to reduce the barriers of talking about MPV to an important other or health care professional, and the barrier of making an appointment.

**Figure 1:**
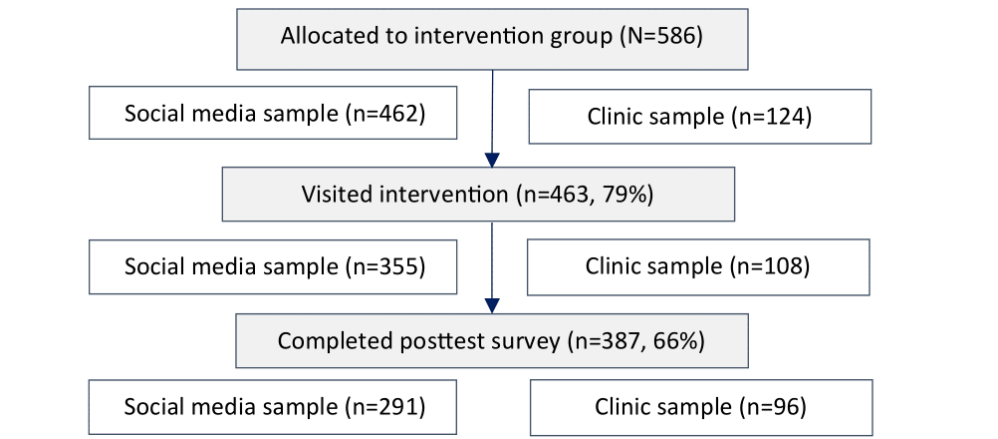
Flow diagram of the recruitment and response of study participants from in the intervention condition.

**Figure 2:**
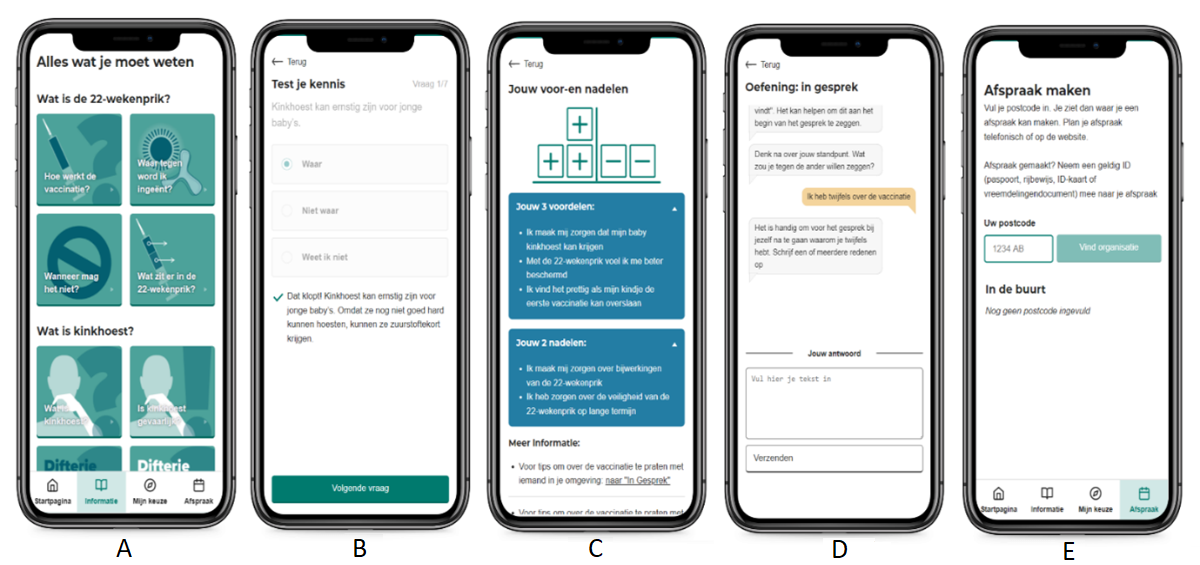
Screenshots of a selection of the web application components. (A) An overview of the information topics. (B) An example of a “test your knowledge” question and tailored feedback. (C) An overview page of the decisional balance with tailored pros and cons. (D) The “prepare a conversation” exercise. (E) The “make an appointment” page.

The decision aid consists of 3 components: an information component, a “my choice” component, and a “make an appointment” component. The information component stimulated active learning by providing information in 3 modes (video, text, and audio) and by providing knowledge questions with immediate feedback. Information topics included “How does MPV work?” “What is in the vaccine?” “What is whooping cough?” “Side effects” “Safety” “When can you not get MPV?” and practical information about MPV, and each page contained on average 94 words in text. The “my choice” component was divided into 3 subcomponents. The first, “test your knowledge,” used active learning and feedback to provide the most basic and relevant information about the vaccine [18-21]. The second, “weighing pros and cons,” aimed to improve decisional certainty by asking participants to fill out questions about potential considerations and then providing a tailored overview of the participants’ answers using a decisional balance [22]. The questions in the “test your knowledge” and “weighing pros and cons” components can be found in the Results section and Figure 1. The third component is “prepare a conversation about the vaccine.” In this chat-like conversational component, the participants prepared for a conversation with a significant other, indicating what they wanted to gain from a conversation with an important social referent or health care provider, and what their feelings, needs, and questions were regarding the MPV. At the end of the exercise, they could save an overview of their answers. Fourth, the “make an appointment” component aimed to decrease the barrier to getting an MPV by providing a postcode-based location finder for an MPV appointment. Participants could navigate through the web application using a menu at the bottom of the web page (Figure 2A). Figure 2 shows screenshots of the decision aid.

### Measurements

#### Program Reach

Program reach is a measure of intervention participation that reflects the proportion of participants who used the intervention as a percentage of those who were offered the intervention (ie, the intervention group) [23]. Intervention reach was measured according to computer logs.

#### Program Use

Program use refers to how much the intervention was used by those who reached it. Oftentimes, this is conceptualized either as implementation or the delivered and received dose of the intervention, or both [23]. Because our specific intervention was designed in a way that participants could use the components that were most relevant to them, we will not focus on complete use of the intervention as an outcome. Instead, we will describe the use of the intervention, so it becomes clear which parts of the intervention have contributed to the effects [13]. Intervention use was measured according to computer logs (objectively) and in the survey (subjectively). Every time a participant used the intervention, a record was created linked to their assigned unique participant number. Every time a participant clicked on a page, a question, or a menu option, it was logged, creating a record of which components and pages were visited, as well as what answers were given to questions in interactive components.

Time spent on the intervention was calculated by taking the sum of the difference in time between each event that was logged for a participant. When a participant did not click on anything for more than 4 minutes, the session was considered ended. We chose this cutoff value because some pages take time to read, or to watch a video on, and inactivity for up to 4 minutes is to be expected there. We measured the number of times participants visited the intervention by looking at how often they revisited the intervention after having not used it for at least 10 minutes.

#### Sociodemographics and Determinants of MPV Uptake

Sociodemographics and determinants of MPV were measured with a survey at baseline (<18 wks of pregnancy) to assess the relationship between reach and use with sociodemographics and determinants of MPV uptake.

Age, highest completed educational level, country of birth of the participant, and whether the participant had children and religious affiliation were measured at baseline. The educational level was classified as low (less than secondary or vocational education), intermediate (secondary and vocational education), or high (higher or university education). The country of birth was divided into 2 categories: the Netherlands or other.

Determinants of MPV were measured with a survey at baseline (<18 wks of pregnancy). To measure the determinants targeted in the intervention, we computed composite scores (Table 1). Determinants included MPV intention, attitude about MPV, perceived susceptibility and severity of pertussis for babies, beliefs about the safety and effectiveness of MPV, moral and injunctive norms, affect around MPV, and perceived control. To assess internal consistency, Cronbach alpha was used for scales consisting of more than 2 items, and the Pearson correlation coefficient (*r*) was used for scales consisting of 2 items. Negatively formulated items were reverse-coded (marked with an R in Table 1). The determinants and their measurements were based on an earlier study of the determinants of MPV uptake intention in the Netherlands [24].

**Table 1:** Overview of outcome measures, their scores or scales, and internal consistencies.

Measures and items				Score or scale		Cronbach alpha or Pearson *r*	
**Knowledge (7 items)**				Sum score of correct answers. 0=low knowledge about MPV to 7=high knowledge about MPV (total number of correct answers)		—^a^	
	The MPV^b^ is meant to protect the baby						
	A painful arm is a common side effect of MPV						
	Whooping cough is never serious for young babies (R)^c^						
	The MPV only protects against whooping cough, and not against other diseases (R)^c^						
	After getting MPV, the baby can skip their first vaccination after birth						
	Whooping cough can be transmitted by coughing						
	The MPV protects only my baby, and not me, against whooping cough (R)^c^						
**Decisional certainty** **(3 items)**				1=low decisional certainty to 5=high decisional certainty		0.93 (α)	
	It is clear to me what the best decision for me is regarding the MPV						
	I feel certain about my decision about MPV						
	I find it easy to make a decision about MPV						
**MPV intention (3 items)**				1=low intention of getting MPV to 5=high intention of getting MPV		0.98 (α)	
	I plan to get MPV						
	I expect to get MPV						
	It is probable that I will get MPV						
**Attitude about MPV (4 items)**							
	**I find MPV:**		1=negative to 5=positive		0.93 (α)		
		very bad – very good					
		very unimportant – very important					
		very undesirable – very desirable					
		very unnecessary – very necessary					
**Perceived susceptibility of pertussis (1 item)**							
	The chance that my baby will get whooping cough (without the MPV) is ….		1=very small to 5=very big		—		
**Perceived severity of pertussis (2 items)**							0.62 *(r)*
	Whooping cough in a baby is ...		1=not serious to 5=very serious				
	If your baby gets whooping cough, how likely do you think it is that it will have serious effects?		1= not likely at all to 5= very likely				
**Beliefs safety of MPV (3 items)**				1=very unsafe to 5=very safe		0.71 (α)	
	MPV has been sufficiently tested in pregnant women						
	MPV is safe for pregnant women						
	MPV is safe for the baby						
**Beliefs effectiveness of MPV (3 items)**				1=very ineffective to 5=very effective		0.78 (α)	
	MPV decreases the chance of whooping cough in babies						
	Babies are better protected against whooping cough with MPV than without MPV						
	There is less whooping cough in babies because of MPV						
**Moral norm (2 items)**				1=low moral norm to 5=high moral norm		0.59 *(r)*	
	I find it my responsibility to get vaccinated for whooping cough						
	I find it the responsibility of pregnant women to get MPV						
**Injunctive norms (2 items)**				1=low injunctive norm to 5=high injunctive norm		0.44 *(r)*	
	I think my partner wants me to get an MPV						
	I think my obstetric care provider wants me to get MPV						
**Affect about MPV (5 items)**							
	**About the MPV I feel:**		1=negative to 5=positive		0.89 (α)		
		Comfortable					
		Relaxed					
		Angry (R)^c^					
		Certain					
		Scared (R)^c^					
**Perceived control (5 items)**							
	**I can manage the following situations:**		1=low perceived control to 5=high perceived control		0.83 (α)		
		Assessing information about MPV					
		Having a conversation about MPV					
		Making a decision about MPV					
		Making an appointment to get MPV					
		Going to the Youth Health Centre to get an MPV					

^a^Not available.

^b^MPV: maternal pertussis vaccination.

^c^R: negatively formulated items were reverse-coded.

#### Program Acceptability and Satisfaction

Program acceptability was measured with a post-test survey at 20-22 weeks of pregnancy. We asked participants to evaluate the intervention with a grade (1=very bad and 10=excellent), as well as to rate the different components and characteristics like layout. In addition, participants were asked open-ended questions about which things they valued the most and the least about the decision aid, as well as what could be improved. Table 2 shows an overview of the measures with references. In addition, we added a measure of engagement, given the interactive nature of the intervention.

**Table 2:** Acceptability measurements.

Measures and items						Score or categories			Reference
**Interest**									
	**I found this online decision aid:**								
		Interesting			1 = completely disagree – 5 = completely agree			[25]	
**Usefulness**									
	**I found this online decision aid:**								
		Useful			1 = completely disagree – 5 = completely agree			[25]	
**Ease of use**									
	**I found this online decision aid:**								
		Easy to use			1 = completely disagree – 5 = completely agree			[26]	
**Trustworthiness**									
	**I found this online decision aid:**								
		Trustworthy			1 = completely disagree – 5 = completely agree			[25]	
**Attitude toward application**									
	**How good or bad did you find the following components (if you did not use the component, click not applicable)**								[27]
		The speed of the web app			1 = very bad – 5 = very good				
		The lay-out			1 = very bad – 5 = very good				
		The amount of text			1 = very bad – 5 = very good				
		The videos			1 = very bad – 5 = very good				
		The images			1 = very bad – 5 = very good				
		The option to do a knowledge test			1 = very bad – 5 = very good				
		The option to weigh the pros and cons about MPV^a^			1 = very bad – 5 = very good				
		The option to prepare a conversation			1 = very bad – 5 = very good				
		The option to find a location to get the MPV			1 = very bad – 5 = very good				
**Elaboration**									
	How well did you read the information on the information pages?				1 = very superficially – 5 = very attentively			—^b^	
**Information evaluation**									
	**Understandable (R)^c^**								
		**I found the information in the decision aid:**							
			Difficult to understand	1 = completely disagree – 5 = completely agree			[25]		
**Relevance**									
		**I found the information in the decision aid:**							
			Relevant	1 = completely disagree – 5 = completely agree			[25]		
**Quantity**									
		**I found the information in the decision aid:**							
			Much	1 = completely disagree – 5 = completely agree			[25]		
	**Sidedness**								
		**I found the information in the decision aid:**							
			Independent	1 = completely disagree – 5 = completely agree			[13]		
	**Engagement**								
		**I found the information in the decision aid:**							
			The online decision aid made me think about MPV	1 = completely disagree – 5 = completely agree			—		
	**Support**								
		**I found the information in the decision aid:**							
			The online decision aid helped me in my decision about MPV	1 = completely disagree – 5 = completely agree			[13]		
	**Recall**								
		**I found the information in the decision aid:**							
			I can recall the online decision aid well	1 = completely disagree – 5 = completely agree			[13]		
	**Recommend**								
		**I found the information in the decision aid:**							
			I would recommend the online decision aid	1 = completely disagree – 5 = completely agree			[26]		

^a^MPV: maternal pertussis vaccination.

^b^Not applicable.

^c^R: negatively formulated items were reverse-coded.

#### Analyses

Descriptive analyses (percentages, numbers, means, and SDs) were used to describe the intervention use and acceptability. We used chi-square and independent sample *t* tests to test for baseline differences between participants who visited the decision aid and those who did not reach it, despite being offered the intervention. In view of multiple testing, an effect was considered significant when *P*<.004 (Bonferroni corrected α=.05/13 factors, to avoid a type-1 error [28]).

We also investigated the characteristics of the participants who used each component. We did this for the use of the “Information” component, “Weighing pros and cons,” “Knowledge test,” “Conversation preparation,” “Appointment,” and the use of videos (having watched at least one video). Here, we will also report averages and SDs and whether differences are statistically significant based on chi-square and independent sample t-tests, after Bonferroni correction. We checked if acceptability correlates with time spent on the intervention and education level.

Analyses were performed in R (version 4.1.2; R Core Team) [29].

## Results

### Sample Description

Figure 1 shows the flow diagram of participants in the intervention group. Dropout at the posttest survey (at 20-22 wks of pregnancy) was 33%.

Tables 3 and 4 show the sociodemographic characteristics of participants in the intervention condition (N=586). The mean age of the participants was 32.21 (SD 4.04) years (the average age of women in the Netherlands at the moment of giving birth was 31.7 years in 2021 [30]), about half of the participants (274/586, 46.8%) already had a child, and 97.6% (572/586) of participants were born in the Netherlands. The level of education of the participants was high, with 80% being highly educated compared with 60.9% in the general population among women of the average age of 32.32 (SD 4.04) years in our sample [31].

**Table 3:** Baseline characteristics (measured <18 wks of pregnancy) of the study sample, and for participants who visited the intervention versus those who did not (part 1).

Sociodemographic variables			Total sample intervention group (N=586), n (%)	DA visited at least once, (n=463), n (%)		DA not visited (n=123), n (%)		Chi-square (*df*)		*P* value	
**Recruitment channel**									4.49 (1)		.03
	Clinic sample	124 (21.16)			107 (23.11)		17 (13.82)				
	Social media sample	462 (78.84)			356 (76.89)		106 (86.18)				
**Has at least one child**									2.02 (1)		.15
	No	312 (53.24)			254 (54.87)		58 (47.15)				
	Yes	274 (46.75)			209 (45.14)		65 (52.85)				
**Country of birth**									0.91 (1)		.34
	Netherlands	572 (97.6)			450 (97.19)		122 (99.19)				
	Other	14 (2.4)			13 (2.81)		1 (0.81)				
**Highest education completed**									4.06 (1)^a^		.04^a^
	Low	4 (0.7)			4 (0.86)		0 (0)				
	Intermediate	113 (19.3)			80 (17.28)		33 (26.83)				
	High	469 (80)			379 (81.82)		90 (73.17)				

^a^Low and intermediate versus high educational levels were compared.

**Table 4:** Baseline characteristics (measured <18 wks of pregnancy) of the study sample, and for participants who visited the intervention versus those who did not (part 2)

Sociodemographic variables (scale)	Total sample intervention group (N=586), mean (SD)	DA visited at least once (n=463), mean (SD)	DA not visited (n=123), mean (SD)	*t* test^a^ (*df*)	Cohen *d* (95% CI)	*P* value
Age (years)	32.21 (4.04)	32.22 (4.03)	32.18 (4.10)	–0.10 (189.51)	–0.01 (–0.21 to 0.19)	.92
Religion (1-7)	2.06 (1.29)	2.04 (1.29)	2.13 (1.31)	0.70 (189.22)	0.07 (–0.13 to 0.27)	.48
Intention to accept MPV^b^ (1-5)	4.61 (0.73)	4.62 (0.70)	4.56 (0.83)	–0.81 (170.86)	–0.09 (–0.29 to 0.11)	.42
Attitude MPV (1-5)	4.61 (0.65)	4.63 (0.61)	4.54 (0.78)	–1.12 (163.84)	–0.13 (–0.33 to 0.07)	.26
Beliefs safety (1-5)	4.41 (0.81)	4.19 (1.01)	4.21 (1.01)	–2.63 (159.54)	–0.32 (–0.52 to –0.12)	<.01
Beliefs effectiveness (1-5)	4.58 (0.59)	4.61 (0.53)	4.46 (0.78)	–2.03 (152.91)	–0.26 (–0.46 to –0.06)	.04
Perceived severity (1-5)	4.38 (0.66)	4.40 (0.64)	4.34 (0.72)	–0.75 (175.55)	–0.08 (–0.28 to 0.12)	.45
Perceived susceptibility (1-5)	2.63 (0.80)	2.59 (0.77)	2.80 (0.86)	2.53 (178.42)	0.27 (0.07 to 0.47)	.01
Moral norm (1-5)	4.31 (0.82)	4.31 (0.81)	4.30 (0.88)	–0.12 (180.04)	–0.01 (–0.21 to 0.19)	.91
Knowledge (1-5)	4.68 (1.53)	4.70 (1.52)	4.62 (1.57)	–0.53 (187.15)	–0.06 (–0.25 to 0.14)	.60
Perceived control (1-5)	4.67 (0.48)	4.68 (0.46)	4.60 (0.55)	–1.49 (168.62)	–0.17 (–0.37 to 0.03)	.14
Injunctive norm (1-5)	4.09 (0.98)	4.14 (0.93)	3.91 (1.13)	–2.00 (167.86)	–0.23 (–0.43 to –0.03)	.05
Affect (1-5)	4.39 (0.78)	4.40 (0.77)	4.36 (0.82)	–0.46 (184.28)	–0.05 (–0.25 to 0.15)	.65
Decisional certainty (1-5)	4.39 (0.95)	4.39 (0.95)	4.40 (0.96)	0.13 (189.78)	0.01 (–0.19 to 0.21)	.90

^a^Independent sample *t* test.

^b^MPV: maternal pertussis vaccination.

### Program Reach

In total, 463 out of 586 participants (79%) from the intervention group used the decision aid at least once. Tables 3 and 4 show baseline characteristics for the participants who did versus those who did not visit the intervention despite being offered the intervention (as all participants were in the intervention group of the RCT). Intervention reach appeared higher among those in their first pregnancy (7.71% difference, *P*=.15), those recruited via their midwife rather than via social media (9.29% difference, *P*=.03), and those who had completed a higher educational level (8.65% difference, *P*=.04). There were small differences in baseline scores for determinants of MPV uptake. Overall, determinants were slightly more positive toward MPV among those who visited the intervention. The largest differences in reach of the intervention (difference between users and nonusers) were found for different levels of beliefs about safety (95% CI –0.515 to –0.115; *P*=.009) and perceived susceptibility (95% CI 0.07 to 0.47; *P*=.01), although these differences were not significant after Bonferroni correction.

### Program Use

Table 5 shows the average (SD) time spent on the intervention, the number of times the intervention was visited, the number of clicks, and the number of pages and information pages visited, as well as how much attention the participants indicated having used the decision aid. On average, participants spent 4.25 (SD 4.39) minutes on the decision aid. Most participants used the decision aid once (56.2% of those who used it, 260/586) or twice (123/586, 26.6%). The average number of clicks was 27.24 (SD 25.08) and varied widely. The average number of visited components was 2.70 (SD 1.63), and the average number of information pages was 3.90 (SD 1.36). There was a correlation of 0.75 between the number of sections visited and the number of pages that participants looked at.

Table S1 in Multimedia Appendix 1 shows the number and percentage of participants who visited each component and information page of the intervention. Of the participants who clicked at least one page, 68.8% (319/463) visited at least one information page. The information pages about how MPV works, the side effects, and safety were the most visited information pages. The appointment component, where participants could use a postcode-based location finder, was visited by 53.1% (246/463) of the participants. Of the “my choice” components, the option to weigh pros and cons was the most used, it was completed by 32.6% (151/463) of the participants. A quarter of the participants (116/463, 25.1%) completed the knowledge test. Only 4 of 463 participants (0.8%) completed the conversation preparation.

We investigated the baseline characteristics of participants who used the different intervention components. There were no significant differences after Bonferroni correction.

**Table 5:** Program use: average (SD) time spent on the intervention, the number of clicks, and the number of pages and information pages visited (N=463).

	Mean (SD)	Range (minimum-maximum)
Time spent (mins)	4.25 (4.39)	>0-29.4
Number of times visited (a new “time” was defined by a revisit after at least 10 mins of no activity)	1.86 (1.48)	1-9
Clicks	27.24 (25.08)	1-167
Number of components visited out of the main components (“Homepage,” “Information,” “Appointment,” and interactive “My choice” subcomponents: “Weighing pros and cons,” “Knowledge test,” “Conversation preparation”)	2.70 (1.63)	1-6
Number of information pages visited	3.90 (1.36)	1-19

### Acceptability

Overall, participants evaluated the intervention positively; they gave the intervention an average grade of 8 on a 10-point scale (1=very bad, 10=excellent). This score did not correlate with time spent on the intervention (*r*=.055). Table S2 in Multimedia Appendix 2 shows the mean and SD scores of the subjective evaluations. Mean scores were larger than 4 on a 5-point scale (1=not at all to 5=very much) for whether the decision aid was informative (mean 4.56, SD 0.83), trustworthy (mean 4.5, SD 0.73), useful (mean 4.45, SD 0.74), easy to use (mean 4.27, SD 0.9), and interesting (mean 4.23, 0.74). The speed of the web app, layout, text amount and images, and the option to weigh the pros and cons all received high mean scores (means>4) from participants. Videos, the knowledge test, the option to prepare a conversation, and the option to find a location to get MPV received scores between 3 and 4 and had larger SDs (>1.85). Participants indicated finding the information relevant (4.51). Opinions were divided about whether the information was much (mean 2.68, SD 1.14 with 1=not much and 5=very much), and independent (mean 3.71, SD 1.03). To the question of whether the decision aid helped the participant in their choice about MPV, 38.9% (107/275) answered “agree a bit” or “completely agree.” The mean score was 2.96 (SD 1.38). In addition, 71% (192/270) answered “agree a bit” or “completely agree” to whether they would recommend the decision aid to others (mean score 3.85, SD 0.87).

Because our sample was relatively highly educated, we checked for differences in acceptability between the highest completed educational levels. The only significant difference (*P*<.05) was found in the evaluation of the knowledge test: low and intermediate-educated participants evaluated the knowledge test more positively (mean 4.28, SD 1.53) than high-educated participants (mean 3.81, SD 185).

In the open-ended questions in the survey, participants indicated enjoying the information, the interactive and visual elements such as the videos, and the option to weigh the pros and cons of MPV. When asked what they least enjoyed, some participants indicated that the decision aid was quite long. Answers showed that the needs for depth and amount of information varied among participants. Some indicated wanting more information, while others said it was already too much and quite difficult to follow. Some participants indicated finding it complex to navigate through the decision aid. Many participants also indicated wanting an easier way to make an appointment online. This could not be included in the decision aid because it is organized at the regional level in the Netherlands.

## Discussion

### Reach

This study describes the process evaluation of an online decision aid to promote IDM about MPV. We found that the reach of the intervention was adequate (79%) among our sample. This is slightly higher than in other studies about online decision aids. In other studies, a decision aid for HPV vaccinations was used by 62.8% of participants [13], and 64% of participants used a web-based intervention aimed at healthy dietary and physical activity behaviors [14]. Many studies about similar interventions did not report reach, making it difficult to compare our results. It should be noted here that our sample was selective, with highly educated people overrepresented, despite using 2 methods of recruitment (online recruitment and via midwifery clinics). Therefore, the intervention did not reach the intended diversity of users that we anticipated in the user-centered design. We will go into selection bias and recruitment challenges further under the Strengths and Limitations section.

Those recruited for the study by their midwife more often reached the decision aid than those recruited via social media. In addition, participants who scored higher at baseline on the belief that MPV is safe, and with lower perceived susceptibility of their baby getting pertussis, more often visited the intervention than those who did not believe MPV was safe and those with a higher perceived susceptibility of their baby getting pertussis. However, these differences were not significant after correction for multiple testing. A potential difference may be explained by those who believe that MPV is safe possibly have more faith in a decision aid made by a scientific institute. There were no other significant baseline differences between those who did and did not use the decision aid, although data indicated that use may be higher among those in their first pregnancy.

### Use

Time spent on the intervention (mean 4.25, SD 4.39 minutes) was lower than in the study with the HPV decision aid (20 minutes; [13]). Other studies found similar times of use as we did [32,33]. Time spent on an online intervention, especially in a noncontrolled environment, is difficult to interpret because it is unclear whether participants are doing other things while using the intervention, or whether they spend all the time that is being logged on the intervention. We found a large variance (SD 4.39) in the time used for the intervention, indicating that some participants spent a very short time, and others a longer time on the intervention. How much time is spent on a decision aid may depend on different aspects, such as the total amount of content in the decision aid and the level of doubt about the decision experienced among users. The big difference between 20 minutes spent on the HPV decision aid and the 4.25 minutes spent on our decision aid indicates that the use of our decision aid leaves room for improvement, for example, by keeping the user engaged. In general, 4.25 minutes on average still gives a participant time to absorb some information. The average number of words on an information page was 94, given that Dutch adults read on average 202 words per minute [34]. In light of exercises such as “weighing the pros and cons,” more time is expected to be spent in order to give deliberated answers to the questions. However, there was a wide range of time spent on the intervention (0-29.4 minutes), indicating that some people engaged a lot more than others. A high correlation (*r*=0.75) between the number of pages visited and the time spent on the intervention indicated that those who visited more components of the intervention needed and took more time to absorb the information.

All components of the intervention were used by at least some participants. The most visited components of the decision aid were the information pages about how MPV works, side effects, and the safety of MPV, as well as the component where one could find a location to make an appointment to get MPV, and where the pros and cons of MPV could be weighed. The least used component was the conversation preparation about MPV, and the information pages about secondary issues, such as who is involved in MPV and whether MPV is painful. These outcomes are in line with what we know about decision-making about MPV from earlier studies. These showed that participants have a need for information about safety and side effects [35,36]. The low use of conversation preparation can be explained by that only a specific group of people with low decisional certainty and important others in the environment who potentially disagree with their stance have a need for conversation preparation. However, we did not find major differences in baseline characteristics between those who used specific components and those who did not. The use of videos was overall low. Furthermore, Readspeaker (having text read out loud) was used only by 5 participants and they were all highly educated. Because of the low use of Readspeaker and the low number of low-educated participants in our sample, it is difficult to draw conclusions about the use of this feature. We expected that Readspeaker would mostly be used by low-literacy people, people with dyslexia, and people with other reasons for wanting or not being able to read easily, such as impaired vision. Even though an application like Readspeaker is only useful for a limited group, it is important that decision aids are accessible and inclusive [37]. Because people have different preferred modes of receiving information [32], the fact that video and Readspeaker functions were only used by subgroups is not surprising.

### Acceptability

Participants evaluated the decision aid in general positively, with an overall grade of 8.0 out of 10. They found the decision aid relevant and easy to use. Opinions varied about the videos, the conversation preparation, and the amount of text. This confirms that the needs among participants differ, and therefore some people would have liked more or less text, or did not evaluate all the components as useful. Of those who used the decision aid, 38.9% indicated that it helped them to an extent to decide about MPV and 71.1% would recommend it to others. High acceptability was also found in another study [13]. We believe that extensively involving the target group in the design of the decision aid and fine-tuning it to their needs contributed to high acceptability rates [15].

Participants evaluated the component with a postal code–based location finder for MPV with 3.53 on a scale of 1-5. Upon finding the right location using our decision aid, the procedure for making the appointment varies per region of the Netherlands. This may have caused confusion, and unfortunately, it limits the options within the decision aid for making an appointment. In general, this emphasizes that making an appointment to get an MPV should be made as easy as possible. Furthermore, some participants in the subjective evaluation indicated that the navigation through the decision aid could be improved.

Online health interventions and decision aids are known to have limited reach among lower-educated populations [38,39]. However, the limited number of low-educated and intermediate-educated groups in our sample evaluated the decision aid just as positively as high-educated participants. It is important to research acceptability further among low-educated users, because this study did not include enough low-educated participants to draw firm conclusions on this.

### Strengths and Limitations

A limitation of the study is that we had a selective sample in which highly educated women who were born in the Netherlands were overrepresented. High baseline levels of determinants of MPV uptake further indicate that our sample was subject to selection bias [8]. Caution should be exercised when interpreting the generalizability of this study’s findings, meaning that the reach and use of the intervention are described for the specific population we were able to include in the study. This kind of selection bias is not uncommon in studies about vaccination uptake [40], but it is a problem because interventions are most needed among those in doubt and holding ambivalent beliefs about vaccinations. Although efforts should always be made to make decision aids and other online interventions as suitable as possible for a broad target group, some interventions could also be developed in parallel to meet the needs of specific subgroups, as one approach that fits all may not be realistic. For example, alongside the decision aid, we have developed an antenatal group-care intervention in which MPV is discussed [15]. Unfortunately, the effects and use of this intervention could not be included in the RCT due to COVID-19 regulations at the time of data collection.

In our sample, the attitude and intention toward MPV were already high at baseline. This indicates that the sample included relatively many people who were already positive about MPV, and perhaps more open to using a decision aid than people who are less positive about MPV. However, we found no differences in reach and use of the intervention in our sample based on attitude or intention toward MPV at baseline. In addition, our sample was far more highly educated (80% highly educated) than the average in the Netherlands (30% highly educated) [31]. This means that our conclusions about reach and use of the decision aid have limited generalizability to the general population, and low-educated people may need to be targeted specifically for the decision aid, and studies about similar interventions to reach them. This could, for example, be done using more outreaching methods of recruitment and dissemination, such as was done for the COVID-19 vaccination hotline in the Netherlands [33]. In addition, key figures in communities can be used to disseminate interventions [40].

We found no significant baseline differences regarding determinants of MPV uptake between those who did and did not use the decision aid upon being offered to do so. Because of multicollinearity between determinants and unequal group sizes, we did not perform a multivariate analysis to assess variance between those who did and did not use the intervention, but only assessed differences on the individual determinant level.

### Conclusions

The decision aid had adequate reach, with 79% of the intervention group using the intervention to some extent. Efforts should be made to reach low-educated populations and those who are not already positive about MPV, as this could be improved. This could be done by using a recommendation to use the decision aid from a trusted healthcare professional, or by using a snowball recruitment method [41]. The use of the intervention varied across participants, indicating a variety of needs among the target group. Usage, such as time spent on the intervention, left room for improvement. Acceptability of the intervention was high, indicating that the user-centered design paid off.

## Data Availability

The datasets generated during and/or analyzed during this study are not publicly available because participants did not consent to this, but they are registered at Zenodo (registration number 7661309) and are available upon reasonable request.

## Appendix

Multimedia Appendix 1 Programme use: number and percentage of participants who visited the intervention pages per component among those who visited the decision aid at least once (N=463). The pages are ordered per component by the number of participants that visited them.

Multimedia Appendix 2 Subjective evaluation of the intervention at post test among participants in the intervention group who reported that they had visited the intervention to some extent (n=272).

## Abbreviations

IDM: informed decision-making
MPV: maternal pertussis vaccination
NIP: National Immunisation Programme
RCT: randomized controlled trial
