# Supplementary material for: Online Tailored Decision Aid for Maternal Pertussis Vaccination in a Randomized Controlled Trial: Process Evaluation Study
Source: JMIR Form Res. 2025 Jul 8;9:e50709. doi: 10.2196/50709 (PMC12284449; doi:10.2196/50709)
Supplement: Multimedia Appendix 1 [file formative_v9i1e50709_app1.pdf]

Table S1. Programme use: number and percentage of participants who visited the intervention pages per component among those who visited the decision aid at least once (n=463). The pages are ordered per component by the number of participants that visited them.

Component visited	Number of participants and percent of participants who visited at least one page, n (%)
**Homepage**	463 (100)
Video played	117 (25.3)
**Information**	319 (68.8)
**How does MPV[a] work?**	224 (48.3)
Video played	30 (6.4)
Side-effects	190 (41.1)
**Safety**	166 (35.9)
Video played	10 (2.1)
**When can you not get MPV?**	115 (24.9)
Video played	5 (1)
What is whooping cough?	98 (21.1)
What is in the vaccine	97 (20.9)
Others' experiences	76 (16.4)
What is MPV for?	70 (15.1)
When is MPV given?	70 (15.1)
Is MPV free?	64 (13.9)
What is tetanus?	55 (11.8)
Who administers MPV?	55 (11.8)
**How is MPV administered?**	53 (11.4)
Video played	4 (0.8)
Why was MPV introduced?	50 (10.8)
Why does my child need more vaccines?	48 (10.4)
What is diphtheria?	47 (10.2)
Is whooping cough dangerous?	44 (9.5)
Is MPV painful?	43 (9.3)
Who are involved?	34 (7.3)
**My choice**	241 (52)
**Weighing pros and cons**	169 (36.5)
Completed	151 (32.6)
**Knowledge test**	121 (26.1)
Completed	116 (25.1)
**Conversation preparation**	55 (11.8)
Completed	4 (0.8)
Appointment (postcode location finder)	246 (53.1)
Readspeaker used	5 (1)
