# Supplementary material for: Online Tailored Decision Aid for Maternal Pertussis Vaccination in a Randomized Controlled Trial: Process Evaluation Study
Source: JMIR Form Res. 2025 Jul 8;9:e50709. doi: 10.2196/50709 (PMC12284449; doi:10.2196/50709)
Supplement: Multimedia Appendix 2 [file formative_v9i1e50709_app2.pdf]

Table S2. Subjective evaluation of the intervention at post test among participants in the intervention group who reported that they had visited the intervention to some extent (n=272)

Measures and items		Mean (SD)
Interest		4.23 (0.74)
Usefulness		4.45 (0.74)
Ease of use		4.27 (0.90)
Trustworthiness		4.50 (0.73)
**Attitude toward application**		
	The speed of the web app	4.54 (1.04)
	The lay-out	4.28 (0.99)
	The amount of text	4.45 (0.97)
	The videos	3.62 (1.89)
	The images	4.64 (0.70)
	The option to do a knowledge test	3.81 (1.85)
	The option to weigh the pros and cons about MPV	4.03 (1.59)
	The option to prepare a conversation	3.18 (2.11)
	The option to find a location to get the MPV	3.53 (2.01)
Elaboration		3.53 (1.12)
**Information evaluation**		
	Understandable (R)	1.47 (0.90)
	Relevance	4.51 (0.70)
	Quantity	2.68 (1.14)
	Sidedness	3.71 (1.03)
	Engagement	2.78 (1.26)
	Support	2.96 (1.38)
	Recall	3.47 (1.14)
	Recommend	3.85 (0.87)
